# Immunogenicity and effectiveness of COVID-19 booster vaccination among people living with HIV: a systematic review and meta-analysis

**DOI:** 10.3389/fmed.2023.1275843

**Published:** 2023-10-09

**Authors:** Meng-Qun Cheng, Rong Li, Zhi-Ying Weng, Gao Song

**Affiliations:** ^1^Department of Reproductive Medicine, The Puer People's Hospital, Pu'er, China; ^2^Department of Pharmacy, The Puer People's Hospital, Pu'er, China; ^3^School of Pharmaceutical Science and Yunnan Key Laboratory of Pharmacology for Natural Products, Kunming Medical University, Kunming, China

**Keywords:** COVID-19 vaccines, booster vaccination, immunogenicity, effectiveness, meta-analysis, systematic review, people living with HIV

## Abstract

**Background:**

The effect of booster vaccinations with the coronavirus virus disease (COVID-19) vaccine on people living with HIV (PLWH) remains unknown. In this study, we aimed to investigate the immunogenicity and effectiveness of booster doses of the COVID-19 vaccine in PLWH.

**Methods:**

Literature research was done through the PubMed, Embase, Cochrane Review, and Web of Science databases up to 4 July 2023. Pooled estimates were calculated and compared using the DerSimonian and Laird method for a random effects model. Randomized control trials and observational studies were both considered for inclusion.

**Results:**

We included 35 eligible studies covering 30,154 PLWH. The pooled immune response rate (IRR) of PLWH after the COVID-19 booster vaccination was 97.25% (95% confidence interval [CI], 93.81–99.49), and similar to healthy control (HC) (risk ratio [RR] = 0.98, 95% CI, 0.96–1.00). The pooled IRR for PLWH with CD4^+^ T-cell counts ≤ 200 was 86.27 (95% CI, 65.35–99.07). For Omicron variants, the pooled IRR for PLWH after booster dose was 74.07% (95% CI, 58.83–89.30), and the risk of IRR was reduced by 10% in PLWH compared with HC (RR = 0.90, 95% CI, 0.80–1.00). The T-cell immune response of PLWH was found to be comparable to HC (*p* ≥ 0.05). Subgroup analyses revealed that mRNA vaccines produced a relatively high IRR in PLWH compared to other vaccines. In addition, the results showed that booster vaccination appeared to further reduce the risk of COVID-19-related infections, hospitalizations, and deaths compared with the primary vaccination.

**Conclusion:**

It was shown that booster vaccination with the COVID-19 vaccine provided a high IRR in PLWH and still produced a desirable moderate IRR in PLWH with a CD4^+^ T-cell count of ≤ 200. Importantly, the humoral and T-cell responses to booster vaccination in PLWH were comparable to HC, and similar results were observed with the SARS-CoV-2 Omicron variant. Our review strongly emphasizes the effect of mRNA vaccine booster vaccination in PLWH on eliciting desirable protective IRR. Furthermore, booster vaccination appears to further reduce the risk of COVID-19 infection, hospitalization, and death in PLWH compared to primary vaccination. However, more evidence is needed to confirm its effectiveness.

## Introduction

The World Health Organization (WHO) has declared the end of the coronavirus disease 2019 (COVID-19) emergency. However, the severe acute respiratory syndrome coronavirus 2 (SARS-CoV-2) variants and the risk of breakthrough infections make the post-pandemic era challenging (1–3). At present, the impact of impaired immunity on the progression of SARS-CoV-2 infection and COVID-19 remains difficult to account for in individuals with immunodeficiency diseases such as people living with HIV (PLWH) (4, 5). Immune perturbations from innate and adaptive immunity, chronic inflammation, accelerated immune senescence, a high prevalence of comorbidities, and sociodemographic factors may contribute to the increased risk of SARS-CoV-2 infection in PLWH compared with HIV-negative individuals (6). A WHO report states that PLWH appears to be a significant independent risk factor for SARS-CoV-2 infection (7, 8). This risk is particularly higher for PLWH with advanced disease, low CD4^+^ T-cell levels, or detectable viremia, as they may be more susceptible to severe COVID-19 and death (9–14).

The current meta-analyses on COVID-19 have mainly focused on primary vaccination, and there is a lack of evidence-based data for booster doses (15–18). In the original COVID-19 cohort studies, particularly in developed countries, comparable clinical outcomes were found in PLWH and HIV-negative individuals (19, 20). In contrast, in other cohort studies, PLWH demonstrated worse outcomes, including higher rates of hospitalization and mortality (7, 21). Timely evaluation of the evidence for booster doses of vaccination is necessary to ensure strategy adaptation for high protection of PLWH. Previous studies have shown (22) that COVID-19 mortality is similar in PLWH and HIV-negative individuals when Alpha, Beta, and Delta variants predominate. However, it has been shown that the immunocompromised population is associated with an increased risk of COVID-19-associated mortality in the face of Omicron variants (23, 24). Providing booster doses to the general population may help overcome this problem (25). However, in PLWH, especially in those individuals with severe immunosuppression, the extent of antibody protection regarding additional doses is unknown.

Evidence suggests that the induction of neutralizing antibody responses alone does not adequately protect the organism during SARS-CoV-2 virus infection, and the involvement of T-cell immune responses is pivotal (26, 27). CD4^+^ T-cell and CD8^+^ T-cell levels and the use of antiretroviral therapy (ART) were shown to be correlates of effective immune responses to influenza or pneumococcal vaccines (28, 29). Recent immunization studies in PLWH have shown that assessment of a third dose of the COVID-19 vaccine elicited a strong humoral immune response but reduced T-cell stimulation compared to healthy control (HC) (30, 31). However, deviations in the CD4^+^ to CD8^+^ ratio may also contribute to the decreased responsiveness of PLWH to SARS-CoV-2 infection (32). Further investigations are needed to elucidate the effect of booster vaccines on T-cell immune responses in PLWH.

In summary, the current data on SARS-CoV-2-infected individuals and PLWH raise the question of whether booster vaccinations with COVID-19 vaccines produce the desired immune response. This is especially relevant when faced with conditions such as severe immunosuppression or the Omicron variant. In this meta-analysis and systematic review, we systematically assessed humoral and cellular immune responses after booster doses of COVID-19 vaccination in PLWH and compared them with HC. We also analyzed clinical effectiveness outcome indicators.

## Methods

### Search strategy

We conducted a systematic review and meta-analysis according to the Preferred Reporting Items for Systematic Reviews and Meta-Analyses (PRISMA) recommendations (33). Systematic literature research was completed through PubMed, Embase, Cochrane Review, and Web of Science, with a search date from inception to 4 July 2023. Search terms included (“COVID-19” or “SARS-CoV-2”), (“vaccine” or “vaccination” or “booster”), and (“HIV” or “acquired immunodeficiency syndrome”). More details of the search strategy are provided in the Supplementary material. Two authors (CMQ and LR) independently reviewed the titles, abstracts, and full text of the articles, and disagreements were resolved by two other researchers (SG and WZY).

### Inclusion and exclusion criteria

The inclusion criteria were as follows: (1) studies that reported on PLWH receiving booster doses (≥3 doses) of the COVID-19 vaccine; (2) observational studies (including case-control and cohort studies), randomized clinical trials (RCTs), and non-RCTs; and (3) studies from which data on immunogenicity (humoral and T-cell immunity) and effectiveness (clinical outcome metrics such as associated infections, symptomatic infections, hospitalizations, and deaths) could be extracted. The excluded studies were as follows: (1) non-original articles, such as reviews, commentaries, and meta-analyses; (2) preprints; (3) studies for which data could not be obtained to calculate immune response rates (IRRs) and risk ratio (RR); and (4) those in which the interval between the booster and primary vaccination was less than 3 months or unspecified.

Definitions of IRR may vary between studies (refer to Supplementary Table S1). Booster vaccination was defined as the completion of the vaccination schedule followed by one or more injections. For multiple articles from the same cohort reporting the same results, we selected those with the largest and most recent studies.

### Data extraction

Two researchers (CMQ and LR) independently extracted data based on a predetermined form in Microsoft Excel. Information collected included first author, year of publication, country, age, booster vaccine type and dose, timing after vaccination, previous vaccinations, interval between booster doses and primary vaccination, CD4^+^ T-cell count, antiretroviral therapy (ART), immunoassay, cellular immune response test, unit, positive response threshold, and effectiveness outcome.

### Risk of bias assessment

We assessed cohort studies and case-control studies using the Newcastle-Ottawa Quality Assessment Scale (34). Two investigators (CMQ and LR) independently conducted the quality assessment. Disagreements were resolved by two additional reviewers (SG and WZY).

### Outcomes of interest

The evaluation of immunological effects included both humoral and cellular immunity.

The primary outcome of humoral immunity evaluation is the immune response rate of neutralizing antibodies (nAb) and other antibodies (such as detection of anti-S protein IgG or anti-RBD IgG). The formula was calculated as the IRR = number of immune responses/number of COVID-19 vaccinations × 100%. The evaluation of cellular immunity mainly includes the level of specific T cells: CD4^+^, CD8^+^, and IFN-γ.

Effectiveness outcomes were assessed primarily in terms of COVID-19-associated infections, hospitalizations, serious illnesses, and deaths.

### Data synthesis and statistical analysis

All analyses were visualized using the STATA 17 statistical software. Pooled estimates were calculated and compared using the DerSimonian and Laird method for a random effects model with a 95% confidence interval (CI) (35). Randomized control trials and observational studies were both considered for inclusion. The statistical heterogeneity of the results was calculated using the *I*^2^ statistic. An *I*^2^ statistic value >50% was considered indicative of substantial heterogeneity (36). We performed subgroup analyses by year of publication, continents, booster vaccine type, time after vaccination, interval between booster doses and primary vaccination, ART, and immunological outcomes. Q-tests were used for subgroup comparisons; variables between subgroups were considered significant when the *p*-value for subgroup differences was < 0.05. The publication bias was studied by visual inspection of the funnel plot symmetry as well as by Egger's test (asymmetry is considered if *p* < 0.05) (37). Sensitivity analysis was performed for outcomes that included more than 10 studies. *p*-values of < 0.05 were considered statistically significant.

## Results

### Characteristics of included studies

**Figure 1:**
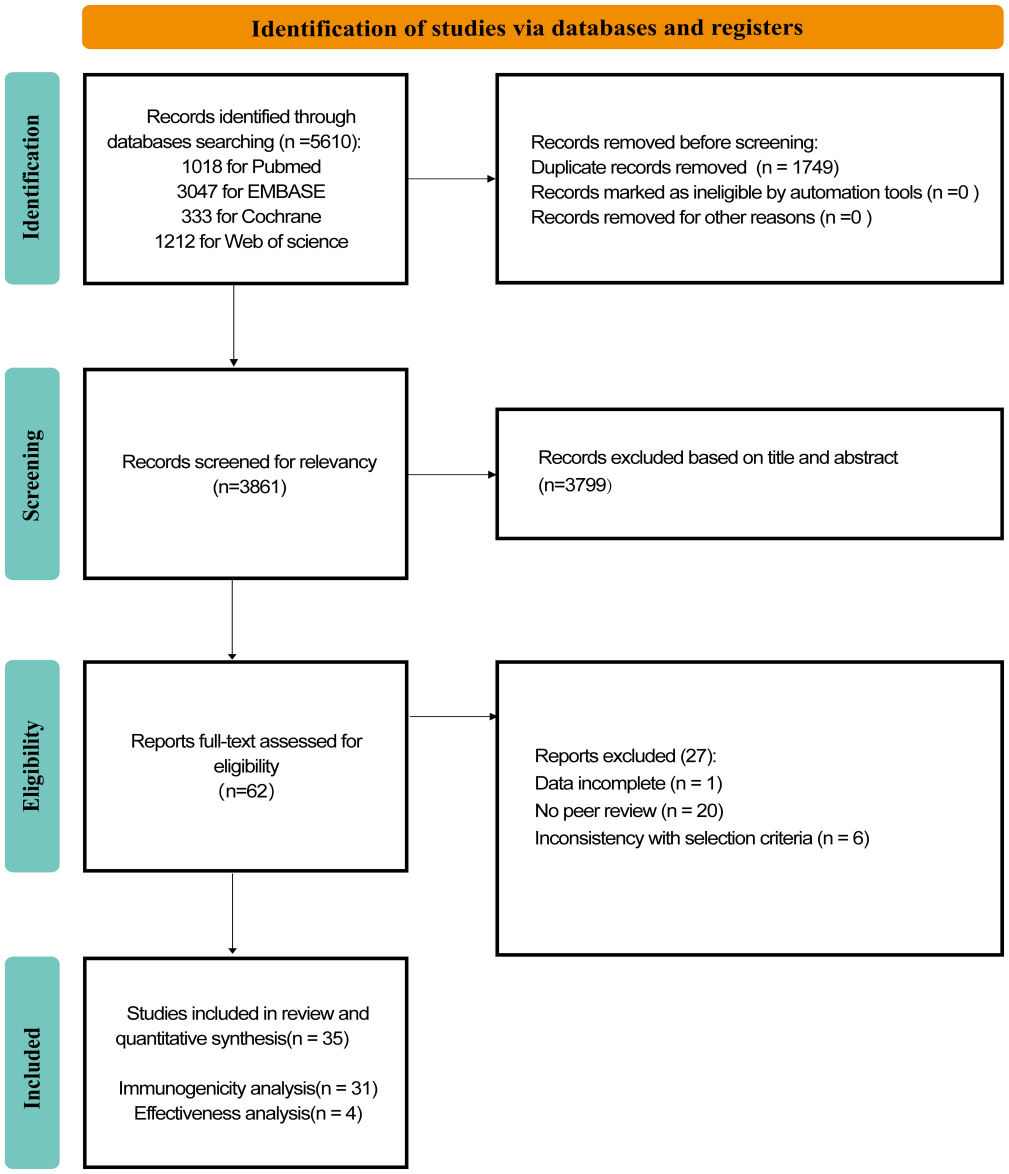
Flowcharts illustrating the article selection process.

In Figure 1, we summarize the selection process, which culminated in the inclusion of 35 studies, of which 31 involved (27, 30, 31, 38–65) immunogenicity assessments (2,652 PLWH) and 4 involved (66–69) effectiveness assessments (27,502 PLWH). Of the 31 immunogenicity studies analyzed (Tables 1, 2; Supplementary Tables S1–S6), all were observational, including 1 case-control study (3.33%) and 30 cohort studies (96.77%). These studies were published in 14 (45.16%) and 17 (54.84%) in 2022 and 2023, respectively. Notably, 9 (29.03%), 17 (54.84%), and 5 (16.13%) studies were conducted in Asia, Europe, and North America, respectively. In addition, 20 (64.52%), 8 (25.81%), and 3 (9.67%) studies were conducted on mRNA vaccine, inactivated vaccine, and multivaccine, respectively. In terms of effectiveness studies, four studies were analyzed. Among the clinical outcome indicators involved, 1 (33.33%) COVID-19-related infection, 1 (33.33%) symptomatic infection, 2 (66.67%) hospitalizations, and 1 (33.33%) death were included (Table 3).

**Table 1:** Immune response rates of PLWH after COVID-19 booster vaccination by subgroup.

	**No. of studies**	**No. of PLWH**	**IRR (%) (95% CI)**	***I^2^*(%)**	***p*_heterogeneity_**	***p_*between groups*_***
Overall	29	2,599	97.25 (93.81–99.49)	93.25	< 0.001	
**Publication year**						0.629
2022	13	1,003	98.25 (95.47–99.87)	76.90	< 0.001	
2023	16	1,596	96.58 (90.34–99.88)	95.64	< 0.001	
**Booster vaccine type**						0.264
mRNA vaccine	18	1,592	98.91 (97.15–99.93)	75.38	< 0.001	
Inactivated vaccine	8	787	92.48 (80.95–99.20)	95.21	< 0.001	
Multivaccine	3	220	98.24 (92.38–100.00)	NR	NR	
**Continents**						0.104
European	16	1,420	98.50 (96.19–99.88)	79.38	< 0.001	
Asian	9	816	93.68 (83.24–99.54)	94.92	< 0.001	
North America	4	363	99.50 (98.09–100.00)	12.80	0.33	
**Time after vaccination**						0.398
≥1 m	22	2,074	96.68 (92.10–99.51)	94.42	< 0.001	
< 1 m	7	525	99.06 (95.82–100.00)	70.53	0.002	
**Interval between booster doses and primary vaccination**						0.231
3–5 m	12	902	98.94 (96.73–100.00)	71.47	< 0.001	
≥6 m	17	1,697	95.77 (89.40–99.54)	95.51	< 0.001	
**Antiretroviral therapy**						0.866
All receiving ART	26	2,415	97.21 (93.56–99.54)	93.62	< 0.001	
Partially receiving ART	3	184	97.37 (77.46–100.00)	NR	NR	
**Immunological outcomes**						0.005
nAb IgG	20	1,655	96.17 (91.03–99.41)	93.59	< 0.001	
Anti-S IgG	7	533	99.17 (96.28–100.00)	72.11	0.002	
Anti-RBD IgG	2	411	100.00 (99.50–100.00)	NR	NR	

PLWH, people living with HIV; IRR, immune response rate; Multivaccine, mRNA or non-mRNA vaccine; m, months; nAb, neutralizing antibodies; S, spike; RBD, receptor binding domain; CI, confidence interval; NR, not reported; ART, antiretroviral therapy; IgG, immunoglobulin.

**Table 2:** Immune response rates of PLWH compared with HC after COVID-19 booster vaccination by subgroup.

	**No. of studies**	**No. of PLWH**	**No. of HC**	**RR (95% CI)**	***I^2^* (%)**	***p*_heterogeneity_**	***p*_subgroup differences_**
Overall	12	1,463	967	0.98 (0.96–1.00)	74.23	< 0.001	
**Publication year**							0.10
2022	6	526	427	1.00 (0.98–1.01)	0	0.62	
2023	6	937	540	0.96 (0.91–1.00)	87.22	0	
**Booster vaccine type**							0.30
mRNA vaccine	6	924	501	1.00 (0.99–1.01)	0	0.73	
Inactivated vaccine	3	319	226	0.88 (0.71–1.08)	92.01	< 0.001	
Multivaccine	3	220	240	0.99 (0.96–1.01)	16.16	0.30	
**Continents**							0.28
European	5	797	424	1.00 (0.99–1.01)	0	0.59	
Asian	4	348	352	0.91 (0.80–1.03)	90.05	< 0.001	
North America	3	318	191	0.99 (0.97–1.01)	0	0.59	
**Time after vaccination**							0.26
≥1 m	9	1,101	823	0.97 (0.94–1.00)	80.19	< 0.001	
< 1 m	3	362	144	1.00 (0.97–1.02)	11.57	0.32	
**Interval between booster doses and primary vaccination**							0.45
3–5 m	4	401	291	0.99 (0.96–1.01)	29.53	0.23	
≥6 m	8	1,062	676	0.97 (0.94–1.01)	81.78	< 0.001	
**Antiretroviral therapy**							0.35
All receiving ART	11	1,341	958	0.98 (0.96–1.00)	76.32	< 0.001	
Partially receiving ART	1	122	9	1.05 (0.91–1.21)	NR	NR	
**Immunological outcomes**							0.10
nAb IgG	10	995	749	0.97 (0.94–1.00)	77.93	< 0.001	
Anti-S IgG	1	122	9	1.05 (0.91–1.21)	NR	NR	
Anti-RBD IgG	1	346	209	1.00 (0.99–1.01)	NR	NR	

PLWH, people living with HIV; HC, healthy control; Multivaccine, mRNA or non-mRNA vaccine; m, months; nAb, neutralizing antibodies; S, spike; RBD, receptor binding domain; CI, confidence interval; NR, not reported; ART, antiretroviral therapy; IgG, immunoglobulin; RR, risk ratio.

**Table 3:** Effectiveness of PLWH after COVID-19 booster dose compared to primary vaccination.

**Author**	**Study design**	**Country**	**Booster vaccine dose**	**Time after vaccination**	**Epidemic of variant strains**	**N_B_/N_P_**	**Effectiveness outcome**	**Effectiveness**
Rasmussen et al. (68)	Cohort	Denmark	3rd	After 14 d	Omicron has been the dominant strain since late December 2021	NR	1) Infection 2) Hospitalization 3) Death	3rd vs. primary vaccination 1) Infection: observed 10% reduced risk [aIRR: 0.9 (0.7–1.0)] 2) Hospitalization: observed 40% reduced risk [aIRR: 0.6 (0.2–1.3)] 3) Death: observed for PWHM 60 years or older 40% reduced risk [IRR: 0.2 (0.1–0.7)]
Rosenthal et al. (69)	Cohort	US	1st boosted	After 14 d	Omicron Predominance (18 December 2021–24 February 2022)	27285/39516	Hospitalizations	1st boosted vs. primary vaccination: 25 vs. 42 (rate %, per 10000)
Biénkowski et al. (66)	Retrospective	Poland	1st boosted	After 4 m	NR	217/217	Symptomatic	1st boosted vs. primary vaccination: 5.5 vs. 5.1 (rate %)
Coburn et al. (67)	Cohort	US	1st boosted	NR	NR	NR/14644	Breakthrough infection	1st boosted vs. primary vaccination Ad26 vs. Ad26: aHR, 0.60 (95% CI, 0.28–1.27) mRNA-1273 vs. mRNA-1273: aHR, 0.50 (95% CI, 0.38–0.67) BNT162 vs. BNT162: aHR, 0.71 (95% CI, 0.58–0.88)

Ad26, Ad26.COV2.S vaccines; BNT162, BNT162 mRNA vaccines; PLWH, people living with HIV; IQR, interquartile range; m, months; w, weeks; d, days; third, rd; first, st; N_B_, number of PLWH for booster vaccine; N_P_, number of PLWH for primary vaccine; NR, not reported; aIRR, adjusted incidence rate ratios; CI, confidence interval; US, United States.

The risk of bias was assessed for 31 meta-analyzed literature. Of these, 6 (19.35%) studies were assessed as low risk of bias, 15 (48.39%) were assessed as moderate risk of bias, and 10 (32.26%) were assessed as high risk of bias. For detailed information on the results of the risk of bias assessment, refer to Supplementary Tables S7, S8.

### Immune response rates among PLWH

The pooled IRR was 97.25% (95% CI, 93.81–99.49) in 29 studies (27, 30, 31, 39, 41–65) involving 2,599 PLWH recipients of booster vaccination, with a high degree of heterogeneity between studies (*I*^2^ = 93.25%) (Table 1; Supplementary Table S2). However, subgroup analyses showed no significant differences between the groups (*p* ≥ 0.05). The IRRs were relatively lower in inactivated vaccine (92.48%, 95% CI, 80.95–99.20), Asian (93.68%, 95% CI, 83.24–99.54), time ≥1 m after vaccination (96.68%, 95% CI, 92.10–99.51), interval between booster doses and primary vaccination ≥6 m (95.77%, 95% CI, 89.40–99.54), and nAb type (96.17%, 95% CI, 91.03–99.41) (Table 1).

**Figure 2:**
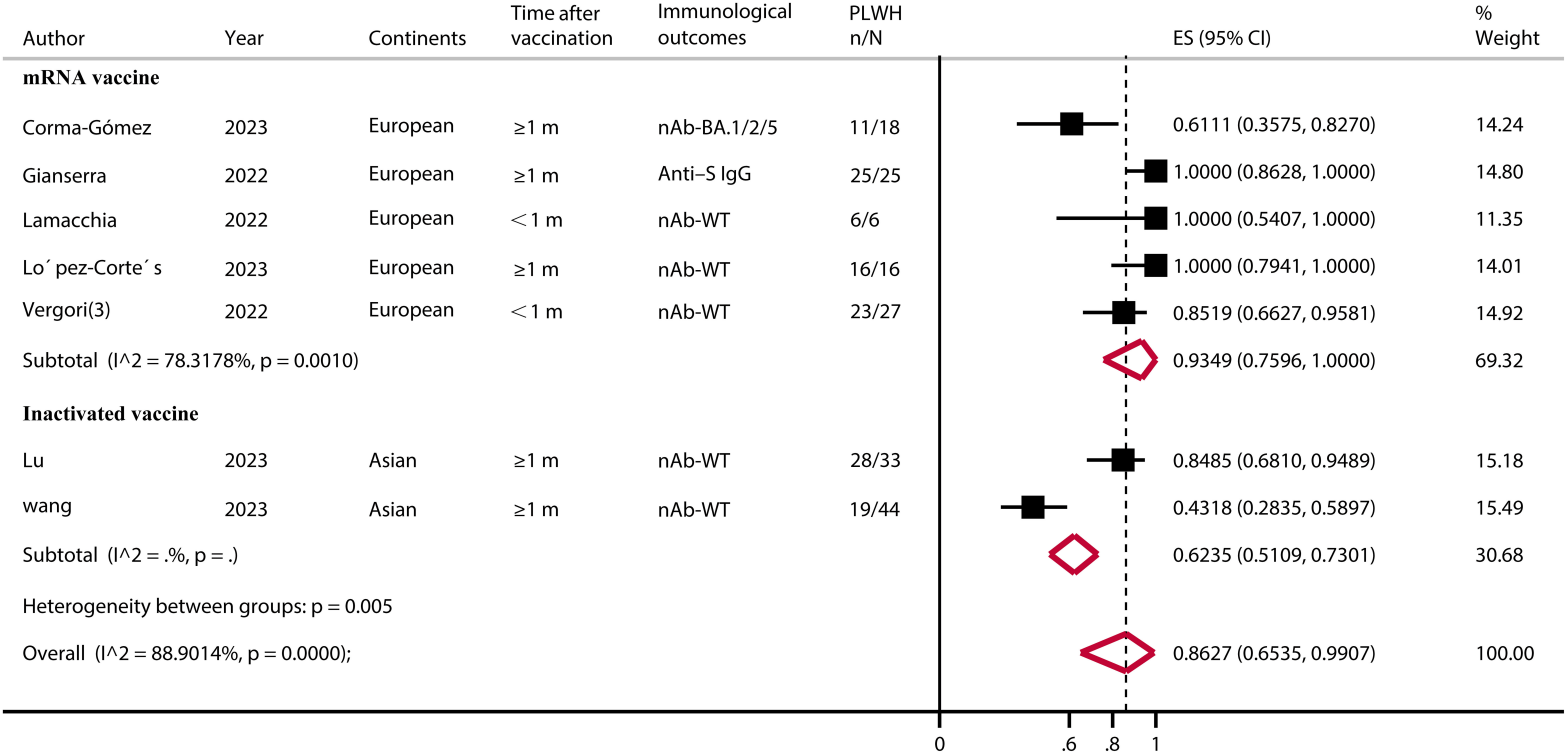
Immune response rate of PLWH (CD4^+^ T-cell count of ≤ 200) after COVID-19 booster vaccination. PLWH, people living with HIV; CI, confidence interval; m, months; ART, antiretroviral therapy; nAb, neutralizing antibodies; S, spike; IgG, immunoglobulin; WT, wild type; ES, effect size (proportions); N, total number of PLWH; n, number of immune responsed PLWH.

A total of seven eligible studies (43, 47, 51, 53, 54, 59, 61) involved PLWH with T-cell counts < 200, none of which had HC. The results showed a pooled IRR of 86.27% (95% CI, 65.35–99.07). A subgroup analysis showed that the IRR was significantly higher for the mRNA vaccine than for the inactivated vaccine (*p* < 0.05) (Figure 2; Supplementary Table S3).

### Comparison of immune responses between PLWH and HC

In 12 studies (27, 30, 31, 44, 50, 52, 54, 56–59, 64), consisting of 1,463 PLWH and 967 HC, there was no significant difference in IRR between PLWH and HC after the booster dose (RR = 0.98, 95% CI, 0.96–1.00). There was a high heterogeneity among the studies (*I*^2^ = 75.90%, *p* < 0.001) (Table 2, Supplementary Table S2). There was no significant difference in IRR between the PLWH and the HC in each subgroup (*p* ≥ 0.05). Of these, the PLWH booster vaccination with mRNA vaccine had the closest degree of immune response to HC (RR = 1.00, 95% CI, 0.99–1.01) and low heterogeneity (*I*^2^ = 0). In addition, a meta-analysis of 6 months after booster vaccination showed a reduction in IRR produced by PLWH compared to the healthy population, but it was not statistically significant (RR = 0.71, 95% CI, 0.35–1.42) (Supplementary Table S4).

### Immune response rates of SARS-CoV-2 Omicron variant among PLWH

**Figure 3:**
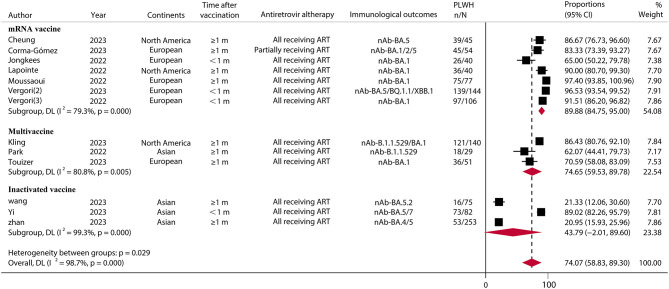
Immune response rates of SARS-CoV-2 omicron variants to PLWH after COVID-19 booster vaccination. PLWH, people living with HIV; CI, confidence interval; m, months; ART, antiretroviral therapy; nAb, neutralizing antibodies; N, the total number of PLWH; n, the number of PLWH with immune responses.

**Figure 4:**
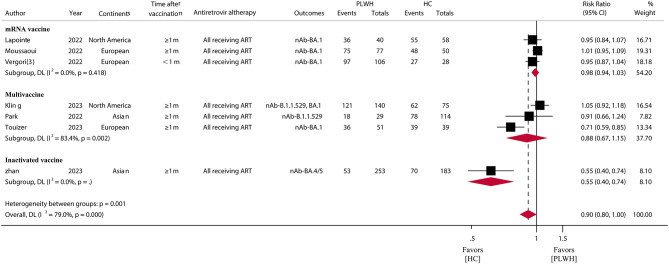
Immune response rates of SARS-CoV-2 omicron variants among PLWH compared to HC after COVID-19 booster vaccination. PLWH, people living with HIV; HC, healthy control; CI, confidence interval; m, months; ART, antiretroviral therapy; nAb, neutralizing antibodies.

For the SARS-CoV-2 Omicron variant, in 13 studies (30, 42, 43, 49, 50, 52, 56, 58–62, 64) involving 1,136 PLWH who received a booster vaccination, the pooled IRR was 74.07% (95% CI, 58.83–89.30) (Figure 3; Supplementary Table S5). The IRR after booster vaccination was similar (RR = 0.90, 95% CI, 0.80–1.00) between PLWH and HC in seven studies consisting of 696 PLWH and 547 HCs. A subgroup analysis of vaccine types showed relatively high IRR and RR for mRNA vaccines (IRR = 89.88%, 95% CI, 84.75–95.00; RR = 0.98, 95% CI, 0.94–1.03) (Figure 4; Supplementary Table S5).

### T-cells immune responses among PLWH

**Figure 5:**
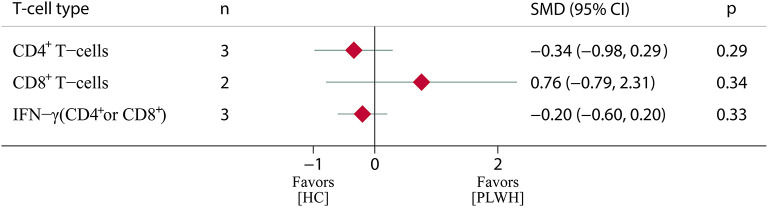
T-cell immune response among PLWH compared to HC after COVID-19 booster vaccination. PLWH, people living with HIV; HC, healthy control; CI, confidence interval; SMD, standardized mean difference; N, number of included studies; *p* < 0.05 represents a statistically significant difference.

In terms of T-cell immune response, a total of six eligible studies (26, 27, 30, 31, 40, 56) were included in the meta-analysis. The results showed that the immune levels of CD4^+^ T cells and IFN-γ were lower in PLWH than in the HC (standardized mean difference (SMD) = −0.34, 95% CI, −0.98 to 0.29; SMD = −0.20, 95% CI, −0.60 to 0.20), but there was no statistically significant difference (*p* > 0.05). The CD8^+^ T-cell levels were higher in PLWH than in the HC (SMD = 0.76, 95% CI, −0.79 to 2.31); however, there was also no statistically significant difference (*p* > 0.05) (Figure 5; Supplementary Table S6).

### Effectiveness of PLWH vaccination between boosters compared to primary

Four studies (66–69), including 27,502 PLWH, reported on vaccine effectiveness following the COVID-19 booster vaccination (Table 3). Of these, three cohort studies and one retrospective study were included. Two study sites were from the United States (US), one from Denmark, and one from Poland. Unfortunately, none were compared with healthy populations. Our results showed that the risk of COVID-19-associated infections, hospitalization, and death appeared to be reduced in PLWH who received a booster dose compared to the primary vaccine.

### Publication bias and sensitivity analysis

Funnel plots and Egger's test indicated a possible publication bias in the meta-analysis of immune response to the original strains after receiving booster doses of the COVID-19 vaccine (test result: *t* = −2.93, df = 11, *p* = 0.0167). To address the issue of publication bias, the trim-and-fill method was used, and the adjusted risk (RR = 1.00, 95% CI, 0.94–1.01) was very close to the original result (RR = 0.98, 95% CI, 0.96–1.00) (Supplementary Figure S1).

To explore the stability of meta-analysis results, sensitivity analysis was performed by excluding each study one by one. For the immune response rate among PLWH compared to HC, after excluding some literature, the results showed that the immune-antibody response of PLWH may be lower than that of the healthy population (Supplementary Figure S2).

## Discussion

This systematic review and meta-analysis provides a comprehensive analysis of the immunogenicity and effectiveness of COVID-19 booster vaccination in PLWH using data from 35 eligible studies and more than 30,000 PLWH individuals with important implications for the field. Our study showed that PLWH had an IRR of 97.25% after COVID-19 booster immunization. It provided a moderate immune response even in PLWH with low CD4^+^ T-cell counts. Importantly, the humoral and cellular immune response to COVID-19 booster immunization was comparable in PLWH compared to HC. The immune response rate was reduced in the face of the Omicron variant but was comparable compared to HC. In terms of effectiveness evaluation, booster vaccination appears to be effective in reducing the risk of COVID-19 infection, hospitalization, and death in PLWH.

There is less evidence-based research regarding booster doses of the COVID-19 vaccine for PLWH, and so far we have found only one study addressing this (18). The meta-analysis study by Zhou et al. (18), which included six studies, calculated a seroconversion rate of 98.4% (95% CI, 94.8–100%) after vaccine boosters for PLWH, and in three of these studies, PLWH had a reduced antibody response compared to the HC (RR = 0.97, 95% CI, 0.94–0.99). Our findings suggest that PLWH vaccinated with vaccine boosters produced humoral immune responses comparable to those of the healthy population (RR = 0.98, 95% CI, 0.96–1.00). However, it is worth noting that the immune response to PLWH may be weaker than that of the healthy population after study-by-study exclusion in stability analyses. In comparison to Zhou et al. (18), our study delved into a more extensive and detailed analysis. We explored various aspects such as subgroup analysis, antibody responses to the Omicron variant, and T-cell immune responses.

In the subgroup analysis, we only found that immunological outcomes may have a significant effect on the pooled immune response rate (*p*_*between groups*_= 0.005). The subgroup analysis of immunological outcomes showed that the immune response rate of nAb was lower than that of other types of antibodies. Previous studies have suggested that inactivated vaccine recipients may be at higher risk of breakthrough infections compared to mRNA vaccines (70, 71). This may also be true in PLWH as our study showed a relatively high immune response to the mRNA vaccine and a relatively weak response to the inactivated vaccine. There is a correlation between the strength of the antibody response and clinical endpoints. In terms of study sites, the pooled IRR was lower in Asia and relatively higher in Europe and North America. This may be due to differences in the type of vaccination, with the majority of vaccines administered in Asian countries being inactivated, but ethnic differences cannot be ruled out (72). A subgroup analysis of vaccination intervals and time to immunoassay after booster showed no significant differences between groups (*p* > 0.05). The results indirectly suggest that vaccine boosters given 3 months after primary vaccination can also produce a relatively favorable immune response. Furthermore, it has been shown that under an effective ART, SARS-CoV-2-infected PLWH have good immune recovery and adequate virological control to generate humoral and T-cell immune responses similar to those of HC (30, 38). In this regard, our findings are similar.

CD4^+^ T-cell counts have been shown to be negatively associated with increased morbidity and mortality in COVID-19 (43, 59). Our results showed that the IRR of PLWH with low CD4^+^ T-cell levels ( ≤ 200 per μl) after vaccine boosters was 86.27% (95% CI, 65.35–99.07). Critically, subgroups showed that the mRNA vaccine still produced a high antibody response (IRR = 93.49%, 95% CI, 75.96–100.00). mRNA vaccines may provide effective antibody protection in severely immunosuppressed PLWH. However, more data support from studies comparing healthy populations is still needed.

Although the emergence of Omicron variants has been associated with a reduction in the severity of COVID-19 (73), recent data suggest that immunocompromised patients are still at high risk of COVID-19 morbidity and mortality, which may be due to the lack of vaccine efficacy in this population (74). Our study showed that vaccine boosters in PLWH provided moderate protection against immune responses in the face of Omicron variants. However, the risk of immune protection was reduced by 10% (RR = 0.90, 95% CI, 0.80–1.00) compared to the healthy population, but there was no statistically significant difference (*p* = 0.05).

While the level of nAb is strongly associated with the protective efficacy of vaccines (75, 76), the massive activation and expansion of antigen-specific CD4^+^ and CD8^+^ T cells are also critical for improving the immune duration and immune memory (77, 78). The immune response of T cells can be divided into two main categories: CD8^+^ and CD4^+^ T-cell responses. CD4^+^ lymphocytes are a group of “helper” T cells that mediate the activity of other immune cells through direct and indirect mechanisms. CD8^+^ T cells, on the other hand, play an important role in attenuating, controlling, and removing intracellular pathogens. Among the included T-cell studies, 3, 2, and 3 studies involved CD4^+^, CD8^+^, and IFN-γ, respectively. The results showed comparable T-cell immune responses in PLWH compared to the healthy population (*p* > 0.05). Of course, this may be attributed to the fact that the included studies were mRNA vaccine-related. Polvere et al. (79) demonstrated that the second vaccination of mRNA vaccine elicits B-cell responses that are quantitatively similar between PLWH and HC, but there are important differences in terms of antibody functionality and phenotypes of memory B cells. More evidence is needed to support changes in B-cell antibody function in PLWH after booster vaccination with COVID-19.

There are fewer studies on the effectiveness of vaccine boosters for PLWH, and no studies with healthy populations as controls were identified, so we included studies comparing only with primary vaccination. It was found that PLWH receiving booster doses appeared to have a reduced risk of COVID-19 infections, hospitalization, and death. Previous studies vaccinating PLWH with influenza (80), hepatitis B (81), and pneumococcal (82) vaccines have demonstrated the presence of a poorer initial antibody response, shorter persistence of the immune response, and more breakthrough infections. The translation of a poorer immune response may have poorer clinical outcomes (70, 75). However, our study showed that booster vaccination has a high and durable immune response in PLWH, and in combination with the currently limited evidence of efficacy, PLWH may benefit from booster vaccinations. However, more research evidence is needed to support this.

There are several limitations to this study. First, the studies included in the literature were observational. Although the researchers attempted to control for potential confounders in the analysis, there may still be unknown confounders that could have affected the results of the study. Furthermore, the use of different immunoassay kits and immunoreactivity thresholds may have biased the results. Predicting protection against COVID-19 has been the subject of debate, and given the paucity of data on clinical efficacy endpoints, nAb levels have recently been recognized as a reliable predictor of protection against COVID-19. In the present study, serum nAb data were preferentially included, and if nAb data were not available, other antibodies, such as Spike-IgG or RBD-IgG antibody data, were included. Additionally, most of the included studies were rated as having a moderate or severe risk of bias, which may affect the reliability and generalizability of the results. Attention also needs to be paid to the presence of instability in the sensitivity analyses, and the results need to be interpreted with caution. Finally, there may be differences in data sources, such as studies eligible for inclusion that were not conducted in Africa, and results from the African PLWH cohort should be interpreted with caution.

## Conclusion

It was shown that booster vaccination with the COVID-19 vaccine provided a high IRR in PLWH and still produced a desirable moderate IRR in PLWH with a CD4^+^ T-cell count of ≤ 200. Importantly, the humoral and T-cell responses to booster vaccination in PLWH were comparable to HC, and similar results were observed with the SARS-CoV-2 Omicron variant. Our review strongly emphasizes the effect of mRNA vaccine booster vaccination on PLWH in eliciting desirable protective IRR. Furthermore, booster vaccination appears to further reduce the risk of COVID-19 infection, hospitalization, and death in PLWH compared to primary vaccination. However, more evidence is needed to confirm its effectiveness.

## Data availability statement

The original contributions presented in the study are included in the article/Supplementary material, further inquiries can be directed to the corresponding authors.

## Author contributions

M-QC: Conceptualization, Data curation, Visualization, Validation, Writing—original draft, Writing—review and editing. RL: Data curation, Formal analysis, Methodology, Validation, Writing—review and editing. Z-YW: Conceptualization, Investigation, Project administration, Resources, Supervision, Writing—review and editing. GS: Conceptualization, Data curation, Formal analysis, Funding acquisition, Investigation, Project administration, Resources, Software, Supervision, Visualization, Writing—original draft, Writing—review and editing.
