# Supplementary material for: Immunogenicity and effectiveness of COVID-19 booster vaccination among people living with HIV: a systematic review and meta-analysis
Source: Front Med (Lausanne). 2023 Oct 9;10:1275843. doi: 10.3389/fmed.2023.1275843 (PMC10591097; doi:10.3389/fmed.2023.1275843)
Supplement: Supplementary file 1 [file Data_Sheet_1.doc]

**Immunogenicity and effectiveness of COVID-19 booster vaccination among people living with HIV: a systematic review and meta-analysis**

**Supplementary Material**

**Literature search strategy**

**Supplementary Table S1. Characteristics of included studies**

**Supplementary Table S2. Humoral immunization of PLWH with COVID-19 vaccine boosters**

**Supplementary Table S3. Humoral immunization of PLWH after COVID-19 vaccine boosters with CD4^+^ T-cell counts less than 200**

**Supplementary Table S4. Immunogenicity of PLWH and HC after COVID-19 booster vaccination after time 6months**

**Supplementary Table S5. SARS-CoV-2-Omicron variant immunogenicity of PLWH and HC after COVID-19 booster vaccination**

**Supplementary Table S6. T-cell immune responses to COVID-19 booster vaccination in PLWH**

**Supplementary Table S7. Risk of bias of all included cohort studies using the Newcastle-Ottawa quality assessment scale**

**Supplementary Table S8. Risk of bias of all included case-control studies using the Newcastle-Ottawa quality assessment scale**

**Supplementary Figure S1. Risk of bias of all included studies**

**Supplementary Figure S2 Sensitivity analysis**

**Supplementary Appendix. The detailed search strategy.**

**Coverage: from the inception to July 4, 2023**

| **Database** | **Result** | **Search strings** |
| --- | --- | --- |
| PubMed | 1018 | #1 COVID-19[Mesh] OR SARS-CoV-2[Mesh] OR "COVID-19 Vaccines"[Mesh] OR COVID-19[Title/Abstract] OR SARS-CoV-2[Title/Abstract]  #2 vaccines[Mesh] OR vaccination[Mesh] OR immunization[Mesh] OR vaccin*[Title/Abstract] OR immuniz*[Title/Abstract] OR booster[Title/Abstract] OR COVID-19 vaccine booster[Title/Abstract] OR booster shot[Title/Abstract]  #3 “HIV Infections” [MeSH] OR “HIV”[MeSH] OR “hiv”[tw] OR hivinfect*[tw] OR “human immunodeficiency virus”[tw] OR “human immunedeficiency virus”[tw] OR “human immuno-deficiency virus”[tw] OR “human immune-deficiency virus”[tw] OR ((human immun*) AND (“deficiency virus”[tw])) OR “acquired immunodeficiency syndrome”[tw] OR “acquired immunedeficiency syndrome”[tw] OR“acquired immuno-deficiency syndrome”[tw] OR“acquired immune-deficiency syndrome”[tw] OR “PLWH”[tw] OR ((acquired immun*) AND (“deficiency syndrome”[tw])) |
| EMBASE | 3047 | ('coronavirus disease 2019'/exp OR 'severe acute respiratory syndrome coronavirus 2'/exp OR 'covid 19':ab,ti OR 'sars-cov-2':ab,ti OR 'sars coronavirus 2':ab,ti OR 'severe acute respiratory syndrome coronavirus 2':ab,ti OR '2019-ncov':ab,ti OR '2019 ncov':ab,ti OR 'ncov-19':ab,ti OR 'ncov 19':ab,ti OR 'coronavirus disease-19':ab,ti OR 'coronavirus disease 19':ab,ti OR 'coronavirus disease 2019':ab,ti OR 'coronavirus disease-2019 virus':ab,ti OR 'ncov':ab,ti OR 'novel coronavirus':ab,ti) AND ('vaccine'/exp OR 'sars-cov-2 vaccine'/exp OR 'vaccination'/exp OR 'immunization'/exp OR 'vaccin*':ab,ti OR 'immunis*':ab,ti OR 'immuniz*':ab,ti OR 'booster':ab,ti OR 'COVID-19 vaccine booster':ab,ti OR 'booster shot':ab,ti) AND 'human immunodeficiency virus infection'/exp OR 'human immunodeficiency virus'/exp OR 'hiv':ti,ab OR 'human immunodeficiency virus':ti,ab OR 'human immuno-deficiency virus':ti,ab OR 'human immunedeficiency virus':ti,ab OR 'human immune-deficiency virus':ti,ab OR 'acquired immune-deficiency syndrome':ti,ab OR 'acquired immunedeficiency syndrome':ti,ab OR 'acquired immunodeficiency syndrome':ti,ab OR 'acquired immunodeficiency syndrome':ti,ab |
| Cochrane Central Register of Controlled Trials | 333 | #1 MeSH descriptor: [COVID-19] explode all trees  #2 MeSH descriptor: [SARS-CoV-2] explode all trees  #3 #1 or #2  #4 ("COVID-19" or "COVID 19" or "SARS-CoV-2" or "SARS Coronavirus 2" or "Severe Acute Respiratory Syndrome Coronavirus 2" or "2019-nCoV" or "Coronavirus Disease-19" or "Coronavirus Disease 19" or "Coronavirus Disease 2019" or "Coronavirus Disease-2019 Virus" or nCoV or "Novel Coronavirus"):ti,ab,kw (Word variations have been searched)  **#5 #3 or #4**  #6 MeSH descriptor: [COVID-19 Vaccines] explode all trees  #7 MeSH descriptor: [Vaccines] in all MeSH products  #8 #6 or #7  #9 (vaccin* or immunis* or immuniz* or inoculation or booster or COVID-19 vaccine booster or booster shot):ti,ab,kw (Word variations have been searched)  **#10 #8 or #9**  **#11** hiv OR ‘hiv infect*’ OR ‘human immunodeficiency virus’ OR ‘human immunedeficiency virus’ OR ‘human immuno-deficiency virus’ OR ‘human immune-deficiency virus’ OR ((human immun*) AND (“deficiency virus”)) OR ‘acquired immunodeficiency syndrome’ OR ‘acquired immunedeficiency syndrome’ OR ‘acquired immunodeficiency syndrome’ OR ‘acquired immune-deficiency syndrome’ OR ((acquired immun*) AND (‘deficiency syndrome’))  **#12 #5 AND #10 AND #11** |
| Web of Science | 1212 | (TS=("COVID-19" OR "COVID 19" OR "SARS-CoV-2" OR "SARS Coronavirus 2" OR "Severe Acute Respiratory Syndrome Coronavirus 2" OR "2019-nCoV" OR "2019 nCoV" OR "nCoV-19" OR "nCoV 19" OR "Coronavirus Disease-19" OR "Coronavirus Disease 19" OR "Coronavirus Disease 2019" OR "Coronavirus Disease-2019 Virus" OR "nCoV" OR "Novel Coronavirus") AND TS=(vaccin* OR vaccination OR immunization OR inoculation OR booster OR COVID-19 vaccine booster OR booster shot) AND (TS=(hiv OR ‘hiv infect*’ OR ‘human immunodeficiency virus’ OR ‘human immunedeficiency virus’ OR ‘human immuno-deficiency virus’ OR ‘human immune-deficiency virus’ OR ((human immun*) AND (“deficiency virus”)) OR ‘acquired immunodeficiency syndrome’ OR ‘acquired immunedeficiency syndrome’ OR ‘acquired immunodeficiency syndrome’ OR ‘acquired immune-deficiency syndrome’ OR ((acquired immun*) AND (‘deficiency syndrome’))))  Indexes=SCI-EXPANDED, SSCI, A&HCI, CPCI-S, CPCI-SSH, BKCI-S, BKCI-SSH, ESCI, CCR-EXPANDED, IC Timespan = All years |

**Supplementary Table S1. Characteristics of included studies.**

| Author | Year | Study design | Country | Age(Years)† | Booster vaccine | Booster dose | Time after vaccination | Previous vaccinations | Interval between booster doses and primary vaccination† | CD4 cell count(per μL)† | Antiretrovir altherapy | Humoral immunity | Cellular immunity |
| --- | --- | --- | --- | --- | --- | --- | --- | --- | --- | --- | --- | --- | --- |
| Alexandrova | 2023 | cohort | Canada | PLWH: 43 (36, 57); HC: 44 (38, 56) | BNT/mRNA-1273 | 3rd | after 1 m | BNT/mRNA-1273/Adenovirus vector | > 3 m | 700 (480-839) | All |  | ✔ |
| Basso | 2022 | cohort | Italy | 55 (48-61) | BNT | 3rd | after 6 m | BNT | > 6 m | 639 (486-845) | All | ✔ |  |
| Bessen | 2022 | cohort | Germany | PLWH (mean ± SD, 46.1 ± 10.9); HC (mean ± SD, 39.4 ± 11.9) | BNT/mRNA-1273 | 3rd | after 4-6 w | BNT/mRNA-1273 | > 3 m | mean: 756.4, range: 79-1562 | All |  | ✔ |
| Chan | 2022 | cohort | China | 49(40-56.5) | Ina | 3rd | ＜90 d (median 33 d, IQR 28-53 d) | Ina | > 3 m | 564.5 (394-733) | All | ✔ |  |
| Cheung | 2023 | observational cohort | Canada | 57(44-65) | BNT/mRNA-1273 | 3rd | after 1 m | ChAdO/mRNA | 182 d(134-192) | 720 (540-920) | All | ✔ |  |
| Corma‐Gómez | 2023 | case-control | Spain | < 200: 56 (52-58); ≥ 200: 58 (54-64) | BNT | 1st booster | after 4-8 w | BNT/mRNA‐1273/ChAdO nCoV‐19/Ad26 | < 200: 6.8 (5.9-7.3); ≥ 200: 5.9 (5.3-6.9) | 163 (34-305)^#^ | 52(96%) | ✔ |  |
| Costiniuk | 2023 | prospective observational cohort | Montreal, Toronto, Ottawa, and Vancouver | PLWH: 54.4 (42.3-62.8) HC: 42.0 (34.0-54.0) | BNT/mRNA-1273/Unknown | 3rd | after 4 w (±2 weeks) | mRNA/ChAdO/Ad26 | 181 d (162-191) | 650 (434-855) | 52(96%) | ✔ |  |
| Fidler | 2022 | cohort | UK | 42.5 (37.2-49.8) | BNT/mRNA-1273 | 2st booster | ＜182 d | ChAdO nCoV-19 | 6-12 m | 694.0 (573.5-859.5) | All | ✔ | ✔ |
| Fusco | 2023 | retrospective observational | Italy | 51 (14) | BNT | 3rd | after 64 d(range 3-169) | BNT | > 3 m | 707(389) | All | ✔ | ✔ |
| Gianserra | 2022 | prospective | Italy | 53 (48-61) | BNT | 3rd | after 4 w | BNT | 5.54 m (5.27-5.50) | 687(488-929)^#^ | All | ✔ |  |
| Hassold | 2022 | observations | France | 56.0 (52.9-66.8) | BNT/mRNA-1273 | 3rd | 2.4 m (1.63-3.0) | BNT/mRNA-1273/ChAdO-nCoV19 | 6 m | 556 (286-726) | All | ✔ |  |
| Heftdal | 2023 | prospective observational cohort | Denmark | PLWH: 56 (48-63) HC: 55 (44-63) | BNT | 3rd | after 4 m | BNT | 6 m | 640 (490-800) | All | ✔ | ✔ |
| Jongkees | 2022 | cohort | Netherlands | 63 (60-66) | mRNA-1273 | 3rd | after 28 d | BNT/mRNA-1273/ChAdO-S/Ad26 | 172 d (154-195) | 650 (423-941) | All | ✔ | ✔ |
| Kling | 2023 | cohort | USA | PLWH(mean(SD), 58.0 (10.1); HC(mean(SD),54.9 (12.4)) | BNT/mRNA-1273/Ad26 | 1st booster | PLWH: 111.5 d (45-153.25); Controls: 109 d(83.5-128) | BNT/mRNA-1273/Ad26 | 6 m | 760.2 (93-1639) | All | ✔ |  |
| Lamacchia | 2022 | prospective | Italy | Mean(SD): 58.5(8.9) | BNT | 2st booster | after 7 d | mRNA-1273/BNT | Mean(SD): 119(29) | Mean(SD): 548.2(430.8)^#^ | 6/8(75%) | ✔ | ✔ |
| Lapointe | 2022 | Retro | Canada | PLWH: 54 (40-61); HC: 47 (35-70) | BNT/mRNA-1273 | 3rd | after 1 m | mRNA/ChAdO | 183 d(143-191) | 715 (545-943) | All | ✔ |  |
| Lo´ pez-Corte´ s | 2023 | prospective cohort | Spain | IR: 48 (42-53); DIR: 56 (47-60) | mRNA-1273/BNT | 3rd | after 1 m | mRNA-1273/BNT | 204 d (175-212) | 654 (512-849)^#^ | All | ✔ |  |
| Lu | 2023 | prospective observational | china | 57 (46-64) | Ina | 3rd | after 1 m | Ina | > 6 m | 362 (227-501)^#^ | All | ✔ |  |
| Malin | 2023 | retrospective cohort | Germany | 55 (44-59) | BNT/mRNA-1273 | 3rd | after 13 w (13-14) | BNT/mRNA-1273 | >22 w | 670 (540-850) | All | ✔ | ✔ |
| Moussaoui | 2022 | non-randomised | Liège (Belgium) | mean ± SD:PLWH: 45.6 ± 10.7; HC: 43.0 ± 10.0 | BNT/mRNA-1273 | 3rd | after 2-8 w | BNT/mRNA-1273/ChAdO | 27 w(25-31) | 680 (546-898) | All | ✔ | ✔ |
| Park | 2022 | prospective observational | Korea | PLWH: 44 (34-56); HC: 35 (26-45) | mRNA/adenovirus-vector | 3rd | at 63 d | mRNA/adenovirus-vector | > 3 m | 670.0 (527.1-830.3) | All | ✔ | ✔ |
| Tan | 2022 | prospective | china | PLWH: 38 (33-47);HC: 32 (30-45) | Ina | 3rd | after 14 d | Ina | 7 m | 542 (422-643) | All | ✔ |  |
| Touizer | 2023 | observational cohort | UK | 46 (21-93) | BNT/mRNA-1273/AZD1222 | 3rd | approximately 7-149 d | BNT/mRNA-1273/AZD1222 | >4 m | 602 (range: 22-1360) | All | ✔ |  |
| Vergori(1) | 2022 | Retro observational cohort | Italy | 54 (47-59) | BNT/mRNA-1273 | 3rd | after 15 d | BNT/mRNA-1273/Ad26/ChAdOx | 142 d | 45 (20-122) | All | ✔ | ✔ |
| Vergori(2) | 2023 | observational cohort | Italy | 56 (47-61) | Bivalent vaccine original/BA.4/5(BNT/mRNA-1273.214) | 3rd booster | approximately 15 d | NR | >3 m | 409 (264-597)^#^ | All | ✔ | ✔ |
| Vergori(3) | 2022 | Retro observational cohort | Italy | PLWH: 45 (42-52); HC: 46.5 (34-53) | BNT/mRNA-1273 | 3rd | after 2 w | NR | 156 d (152-159) | NR^#^ | All | ✔ |  |
| wang | 2023 | observational cohort | china | NR | Ina | 3rd | after 6m/2 m/1 m | Ina | 3/5 m | NR^#^ | All | ✔ |  |
| Yi | 2023 | cohort | china | 30 (19-60) | Ina | 3rd | 28 d (24-32) | Ina | >6 m | 403 (13-827) | All | ✔ | ✔ |
| Zeng | 2023 | cohort | china | 34 (30-39) | Ina | 3rd | after 35 d | Ina | 195(185.5-213) | 522(417.5-674.5) | All | ✔ |  |
| zhan | 2023 | cohort | china | 35 (30-41) | Ina | 3rd | after 14-89 d/ 90-180 d/ >180 d | Ina | > 180 d | 482 (355-700) | All | ✔ |  |
| Zhang | 2023 | cohort | china | NR | Ina | 3rd | after 2 m/1 m | Ina | 3/5 m | ＜500 | All | ✔ | ✔ |

Abbreviations: SARS-CoV-2, Severe Acute Respiratory Syndrome Coronavirus 2; PLWH, people living with hiv; HC, Healthy Control; SD, Standard Deviation; ART, Antiretroviral therapy; ELISA, enzyme-linked immunosorbent assay; RBD, receptor binding domain; Ig, immunoglobulin; nAb, neutralizing antibodies; S, spike; IQR, interquatile range; IR, immunological responders; DIR, discordant immune responders; UK, United Kingdom; NR, Not reported; < 200, CD4^+^ T-cell counts < 200 cells/mm^3^.

† Reported as median (IQR) unless otherwise stated.

# indicates that the study was designed with subgroup data for T-cell counts less than 200.

**Supplementary Table S2. Humoral immunization of PLWH with COVID-19 vaccine boosters**

| Author | Publication year | Continents | Booster vaccine type | Time after vaccination | Interval between booster and primary vaccination | Antiretrovir altherapy | Immunological outcomes | Threshold for positive response | PLWH  (N1) | PLWH  (n1) | HC  (N1) | HC  (n1) |
| --- | --- | --- | --- | --- | --- | --- | --- | --- | --- | --- | --- | --- |
| Basso | 2022 | European | mRNA vaccine | ≥1 m | ≥6 m | All | Anti-S IgG | DiaSorin Liaison IgG titers≥33.8 BAU/mL | 184 | 183 | NR | NR |
| Chan | 2022 | Asian | Inactivated vaccine | ≥1 m | 3-5 m | All | nAb | sVNT≥30% | 122 | 111 | NR | NR |
| Cheung | 2023 | North America | mRNA vaccine | ≥1 m | ≥6 m | All | nAb | speciﬁc nAb live virus assays > 1/20 dilution | 45 | 44 | NR | NR |
| Corma‐Gómez | 2023 | European | mRNA vaccine | ≥1 m | ≥6 m | Partially | nAb | nAb ≥1/20 dilution | 54 | 45 | NR | NR |
| Costiniuk | 2023 | North America | mRNA vaccine | ≥1 m | ≥6 m | Partially | Anti-S IgG | cut off = 1.0; binding antibody units (BAU)/ml | 122 | 122 | 9 | 9 |
| Fidler | 2022 | European | mRNA vaccine | ≥1 m | ≥6 m | All | Anti-S IgG | IgG≥10 EU | 32 | 32 | NR | NR |
| Fusco | 2023 | European | mRNA vaccine | ≥1 m | 3-5 m | All | Anti-S IgG | Antispike IgG>33 BAU/mL | 64 | 63 | NR | NR |
| Gianserra | 2022 | European | mRNA vaccine | ≥1 m | 3-5 m | All | Anti-S IgG | Reactive≥15 AU/mL | 42 | 42 | NR | NR |
| Hassold | 2022 | European | mRNA vaccine | ≥1 m | ≥6 m | All | Anti-S IgG | Antispike IgG>260 BAU/mL | 23 | 17 | NR | NR |
| Heftdal | 2023 | European | mRNA vaccine | ≥1 m | ≥6 m | All | Anti-RBD IgG | positive IgG response>225 AU/mL | 346 | 346 | 209 | 209 |
| Jongkees | 2022 | European | mRNA vaccine | ＜1 m | 3-5 m | All | Anti-S IgG | positivity IgG>33.8 BAU/mL | 66 | 66 | NR | NR |
| Kling | 2023 | North America | Multivaccine | ≥1 m | ≥6 m | All | nAb | nAb Response(sVNT)> 0BAU/mL | 140 | 139 | 75 | 75 |
| Lamacchia | 2022 | European | mRNA vaccine | ＜1 m | 3-5 m | Partially | nAb | cut off=1 BAU/mL | 8 | 8 | NR | NR |
| Lapointe | 2022 | North America | mRNA vaccine | ≥1 m | ≥6 m | All | nAb | nAb>1: 20 dilution | 56 | 55 | 107 | 107 |
| Lo´ pez-Corte´ s | 2023 | European | mRNA vaccine | ≥1 m | ≥6 m | All | nAb | positive inhibition ≥35% | 26 | 26 | NR | NR |
| Lu | 2023 | Asian | Inactivated vaccine | ≥1 m | ≥6 m | All | nAb | >0.15μg/mL | 25 | 25 | 25 | 25 |
| Malin | 2023 | European | mRNA vaccine | ≥1 m | 3-5 m | All | nAb | activity>0 BAU/mL | 76 | 74 | NR | NR |
| Moussaoui | 2022 | European | mRNA vaccine | ≥1 m | ≥6 m | All | nAb | NT50>1:160 | 79 | 77 | 50 | 48 |
| Park | 2022 | Asian | Multivaccine | ≥1 m | 3-5 m | All | nAb | sVNT positive threshold>30% | 29 | 29 | 126 | 126 |
| Tan | 2022 | Asian | Inactivated vaccine | ＜1 m | ≥6 m | All | nAb | nAb titers> 0BAU/mL | 41 | 38 | 18 | 18 |
| Touizer | 2023 | European | Multivaccine | ≥1 m | 3-5 m | All | nAb | 50% inhibitory titers =1: 20 | 51 | 47 | 39 | 39 |
| Vergori(1) | 2022 | European | mRNA vaccine | ＜1 m | 3-5 m | All | nAb | MNA(90)≥1: 10 | 215 | 215 | 98 | 98 |
| Vergori(2) | 2023 | European | mRNA vaccine | ＜1 m | 3-5 m | All | nAb | MNA(90)≥1: 10 | 48 | 48 | NR | NR |
| Vergori(3) | 2022 | European | mRNA vaccine | ＜1 m | 3-5 m | All | nAb | MNA(90)≥1: 10 | 106 | 101 | 28 | 28 |
| wang | 2023 | Asian | Inactivated vaccine | ≥1 m | 3-5 m | All | nAb | MNA≥1: 4 | 75 | 75 | NR | NR |
| Yi | 2023 | Asian | Inactivated vaccine | ＜1 m | ≥6 m | All | nAb | pseudovirus nAb>10 EC50 | 41 | 39 | NR | NR |
| Zeng | 2023 | Asian | Inactivated vaccine | ≥1 m | ≥6 m | All | Anti-RBD IgG | seropositivity S/CO≥1 | 65 | 64 | NR | NR |
| zhan | 2023 | Asian | Inactivated vaccine | ≥1 m | ≥6 m | All | nAb | sVNT positive; Inhibition rate>30% | 253 | 156 | 183 | 159 |
| Zhang | 2023 | Asian | Inactivated vaccine | ≥1 m | ≥6 m | All | nAb | seropositive≥1: 4 | 165 | 133 | NR | NR |

Abbreviations: SARS-CoV-2, Severe Acute Respiratory Syndrome Coronavirus 2; PLWH, people living with hiv; HC, Healthy Control; nAb, neutralizing antibodies; RBD, receptor binding domain; S, spike; m, months; w, weeks; d, days; sVNT, surrogate virus neutralization test; MNA, Microneutralization assay; PRNT, plaque reduction neutralization test; EU, ELISA units; BAU, binding antibody units; AU, arbitrary units; S/CO, signal/cut off; EC50, effective concentration 50%; NT50, neutralises cytopathic effect in 50%; All, All receiving ART; Partially, Partially receiving ART; N1, Total number of PLWH; n1, Number of immuneresponsed PLWH ; N2, Total number of HC; n2, Number of immuneresponsed HC; NR, Not reported.

**Supplementary Table S3. Humoral immunization of PLWH after COVID-19 vaccine boosters with CD4^+^ T-cell counts less than 200**

| Author | Publication year | Continents | Country | Booster vaccine type | Time after vaccination | Interval between booster and primary vaccination | Antiretrovir altherapy | Immunological outcomes | Threshold for positive response | PLWH  (N1) | PLWH  (n1) | HC  (N2) | HC  (n2) |
| --- | --- | --- | --- | --- | --- | --- | --- | --- | --- | --- | --- | --- | --- |
| Corma-Gómez | 2023 | European | Spain | mRNA vaccine | ≥1 m | ≥6 m | Partially | nAb-BA.1, BA.2, and BA.5 strains | nAb≥1/20 dilution | 18 | 11 | NR | NR |
| Gianserra | 2022 | European | Italy | mRNA vaccine | ≥1 m | 3-5 m | All | Anti-S IgG | Reactive≥ 15 AU/mL | 25 | 25 | NR | NR |
| Lamacchia | 2022 | European | Italy | mRNA vaccine | ＜1 m | 3-5 m | Partially | nAb-WT | cut off=1 BAU/mL | 6 | 6 | NR | NR |
| Lo´ pez-Corte´ s | 2023 | European | Spain | mRNA vaccine | ≥1 m | ≥6 m | All | nAb-WT | positive inhibition≥35% | 16 | 16 | NR | NR |
| Lu | 2023 | Asian | china | Inactivated vaccine | ≥1 m | ≥6 m | All | nAb-WT | >0.15μg/mL | 33 | 28 | NR | NR |
| Vergori(3) | 2022 | European | Italy | mRNA vaccine | ＜1 m | 3-5 m | All | nAb-WT | MNA(90)≥1: 10 | 27 | 23 | NR | NR |
| wang | 2023 | Asian | china | Inactivated vaccine | ≥1 m | 3-5 m | All | nAb-WT | MNA≥1: 4 | 44 | 19 | NR | NR |

Abbreviations: SARS-CoV-2, Severe Acute Respiratory Syndrome Coronavirus 2; PLWH, people living with hiv; HC, Healthy Control; nAb, neutralizing antibodies; S, spike; m, months; w, weeks; d, days; WT, wild type; sVNT, surrogate virus neutralization test; MNA, Microneutralization assay; PRNT, plaque reduction neutralization test; EU, ELISA units; BAU, binding antibody units; AU, arbitrary units; S/CO, signal/cut off; EC50, effective concentration 50%; NT50, neutralises cytopathic effect in 50%; N1, Total number of PLWH; n1, Number of immuneresponsed PLWH ; N2, Total number of HC; n2, Number of immuneresponsed HC; NR, Not reported.

**Supplementary Table S4. Immunogenicity of PLWH and HC after COVID-19 booster vaccination after time 6months**

| Author | Publication year | Booster vaccine type | Booster Vaccine dose | Time after vaccination | Previous vaccinations | Outcome | Threshold for positive response | PLWH(N1) | PLWH(n1) | HC(N2) | HC(n2) | Risk ratio (95% CI) |
| --- | --- | --- | --- | --- | --- | --- | --- | --- | --- | --- | --- | --- |
| Heftdal | 2023 | BNT162b2 | 3rd | after 11 m | BNT162b2 | Anti-RBD IgG | IgG response>225 AU/mL | 267 | 267 | 142 | 142 | 1.00(0.99-1.01) |
| wang | 2023 | Ina | 3rd | after 6 m | Ina | nAb-WT | MNA≥1: 4 | 158 | 75 | 13 | 13 | 0.49(0.41-0.60) |
| Overall,(I^2^ = 98.1%, P ＜0 .001) | | | | | | | | | | | | 0.71(0.35-1.42) |

Abbreviations: Ina, Inactivated COVID-19 vaccines; PLWH, people living with hiv; HC, Healthy Control; nAb, neutralizing antibodies; RBD, receptor binding domain; S, spike; WT, wild type; m, months; third, rd; CI, confidence interval; sVNT, surrogate virus neutralization test; MNA, Microneutralization assay; PRNT, plaque reduction neutralization test; EU, ELISA units; BAU, binding antibody units; AU, arbitrary units; S/CO, signal/cut off; EC50, effective concentration 50%; NT50, neutralises cytopathic effect in 50%; N1, Total number of PLWH; n1, Number of immuneresponsed PLWH ; N2, Total number of HC; n2, Number of immuneresponsed HC; NR, Not reported.

**Supplementary Table S5. SARS-CoV-2 omicron variant immunogenicity of PLWH and HC after COVID-19 booster vaccination**

| Author | Publication year | Study design | Continents | Booster vaccine type | Time after vaccination | Interval between booster and primary vaccination | Antiretrovir altherapy | outcomes | Threshold for positive response (unit) | PLWH  (N1) | PLWH  (n1) | HC  (N2) | HC  (n2) |
| --- | --- | --- | --- | --- | --- | --- | --- | --- | --- | --- | --- | --- | --- |
| Cheung | 2023 | cohort | North America | mRNA vaccine | ≥1 m | ≥6 m | All | nAb-BA.5 | speciﬁc nAb live virus assays>1/20 dilution | 45 | 39 | NR | NR |
| Corma-Gómez | 2023 | Case-control | European | mRNA vaccine | ≥1 m | ≥6 m | Partially | nAb-BA.1, BA.2, and BA.5 strains | nAb≥1/20 dilution | 54 | 45 | NR | NR |
| Jongkees | 2022 | prospective | European | mRNA vaccine | ＜1 m | 3-5 m | All | nAb-BA.1 | IgG>33.8 BAU/mL | 40 | 26 | NR | NR |
| Kling | 2023 | cohort | North America | Multivaccine | ≥1 m | ≥6 m | All | nAb-B.1.1.529, BA.1 | nAb Response(sVNT)>0 BAU/mL | 140 | 121 | 75 | 62 |
| Lapointe | 2022 | Retro | North America | mRNA vaccine | ≥1 m | ≥6 m | All | nAb-BA.1 | nAb>1: 20 dilution | 40 | 36 | 58 | 55 |
| Moussaoui | 2022 | cohort | European | mRNA vaccine | ≥1 m | ≥6 m | All | nAb-BA.1 | NT50>1:160 | 77 | 75 | 50 | 48 |
| Park | 2022 | prospective observational | Asian | Multivaccine | ≥1 m | 3-5 m | All | nAb-B.1.1.529 | sVNT positive threshold > 30% | 29 | 18 | 114 | 78 |
| Touizer | 2023 | cohort | European | Multivaccine | ≥1 m | 3-5 m | All | nAb-BA.1 | 50% inhibitory titers=1: 20 | 51 | 36 | 39 | 39 |
| Vergori(2) | 2023 | cohort | European | mRNA vaccine | ＜1 m | 3-5 m | All | nAb-BA.5/BQ.1.1/XBB.1 | MNA(90)≥1: 10 | 144 | 139 | NR | NR |
| Vergori(3) | 2022 | cohort | European | mRNA vaccine | ＜1 m | 3-5 m | All | nAb-BA.1 | MNA(90)≥1: 10 | 106 | 97 | 28 | 27 |
| wang | 2023 | cohort | Asian | Inactivated vaccine | ≥1 m | 3-5 m | All | nAb-BA.5.2 | MNA≥1: 4 | 75 | 16 | NR | NR |
| Yi | 2023 | prospective | Asian | Inactivated vaccine | ＜1 m | ≥6 m | All | nAb-BA.5/BA.7 | pseudovirus nAb>10 EC50 | 82 | 73 | NR | NR |
| zhan | 2023 | cohort | Asian | Inactivated vaccine | ≥1 m | ≥6 m | All | nAb-BA.4/5 | sVNT inhibition rate≥30% | 253 | 53 | 183 | 70 |

Abbreviations: SARS-CoV-2, Severe Acute Respiratory Syndrome Coronavirus 2; PLWH, people living with hiv; HC, Healthy Control; nAb, neutralizing antibodies; m, months; third, rd; sVNT, surrogate virus neutralization test; MNA, Microneutralization assay; PRNT, plaque reduction neutralization test; EU, ELISA units; BAU, binding antibody units; AU, arbitrary units; S/CO, signal/cut off; EC50, effective concentration 50%; NT50, neutralises cytopathic effect in 50%; N1, Total number of PLWH; n1, Number of immuneresponsed PLWH; N2, Total number of HC; n2, Number of immuneresponsed HC; NR, Not reported.

**Supplementary** **Table S6. T-cell immune responses to COVID-19 booster vaccination in PLWH**

| Author | Year | Continents | Booster vaccine | Cellular immune response test | unit | Testing time after 3rd vaccination | PLWH  (N) | T-cell response  (PLWH) | SD  (PLWH) | HC  (n) | T-cell response  (HC) | SD  (HC) |
| --- | --- | --- | --- | --- | --- | --- | --- | --- | --- | --- | --- | --- |
| CD4^+^ T-cells | | | | | | | | | | | | |
| Alexandrova | 2023 | North America | mRNA vaccine | Flow cytometric analysis(Total CD4^+^ %) | Ratio | after 1 m | 38 | 0.0035 | 0.006 | 20 | 0.0029 | 0.004 |
| Bessen | 2022 | European | mRNA vaccine | Flow cytometric analysis(CD4^+^ %) | Ratio | after 4-6 w | 15 | 47.62 | 16.32 | 15 | 62.8 | 5.51 |
| Moussaoui | 2022 | European | mRNA vaccine | QuantiFERON assay(IFN-γ Ag1 CD4^+^) | IU/mL | after 2-8 w | 77 | 1.1 | 1.59 | 50 | 1.3 | 1.65 |
| CD8^+^ T-cells | | | | | | | | | | | | |
| Alexandrova | 2023 | North America | mRNA vaccine | Flow cytometric analysis(Total CD8^+^ %) | Ratio | after 1 m | 38 | 0 | 0.0005 | 20 | 0 | 0.0009 |
| Bessen | 2022 | European | mRNA vaccine | Flow cytometric analysis(CD8^+^ %) | Ratio | after 4–6 w | 15 | 42.82 | 15.02 | 15 | 25.35 | 4.17 |
| IFN-γ (CD4^+^ or CD8^+^ T-cells) | | | | | | | | | | | | |
| Heftdal | 2023 | European | mRNA vaccine | ELISA(IFN-γ) | mIU/mL | after 4 m | 178 | 1174 | 2168 | 135 | 1104 | 2045 |
| Park | 2022 | Asian | Multivaccine | IGRA(IFN-γ) | mIU/mL | at 63 d | 9 | 434.84 | 586.05 | 76 | 484.96 | 364.91 |
| Vergori(1) | 2022 | European | mRNA vaccine | ELISA(IFN-γ) | pg/mL | after 15 d | 204 | 7.36 | 2.9 | 46 | 8.74 | 1.71 |

Abbreviations: SARS-CoV-2, Severe Acute Respiratory Syndrome Coronavirus 2; PLWH, people living with hiv; HC, Healthy Control; SD, Standard Deviation; ELISA, enzyme-linked immunosorbent assay; IGRA, interferon-gamma-release-assay; IFN, interferon; PBMCs, peripheral blood mononuclear cells; AIM, activation induced marker assay; nAb, neutralizing antibodies; IQR, interquatile range; m, months; w, weeks; d, days; Ns, Total number of PLWH; ns, Total number of HC; NR, Not reported.

**Supplementary Table S7. Risk of bias of all included cohort studies using the Newcastle-Ottawa quality assessment scale**

| Author | Year | Selection | | | | Comparability | Outcome | | | Total score | Risk level^#^ |
| --- | --- | --- | --- | --- | --- | --- | --- | --- | --- | --- | --- |
|  |  | Representativenes s of the Exposed Cohort | Selection of the Non-Exposed Cohort | Ascertainment of Exposure | Demonstration That Outcome of Interest Was Not Present at Start of Study |  | Assessment of Outcome | Was Follow-Up Long Enough for Outcomes to Occur | Adequacy of Follow Up of Cohorts |  |  |
| Alexandrova | 2023 | 1 | 0 | 1 | 0 | 2 | 1 | 1 | 1 | 7 | low |
| Basso | 2022 | 1 | 0 | 1 | 0 | 0 | 1 | 1 | 1 | 5 | moderate |
| Bessen | 2022 | 0 | 0 | 1 | 0 | 1 | 1 | 1 | 1 | 5 | moderate |
| Chan | 2022 | 1 | 0 | 1 | 0 | 0 | 1 | 1 | 1 | 5 | moderate |
| Cheung | 2023 | 1 | 0 | 1 | 0 | 0 | 1 | 1 | 0 | 4 | high |
| Costiniuk | 2023 | 1 | 0 | 1 | 0 | 1 | 1 | 1 | 0 | 5 | moderate |
| Fidler | 2022 | 0 | 0 | 1 | 0 | 1 | 1 | 1 | 0 | 4 | high |
| Fusco | 2023 | 1 | 0 | 1 | 0 | 0 | 1 | 1 | 1 | 5 | moderate |
| Gianserra | 2022 | 1 | 0 | 1 | 0 | 0 | 1 | 1 | 1 | 5 | moderate |
| Hassold | 2022 | 0 | 0 | 1 | 0 | 0 | 1 | 1 | 1 | 4 | high |
| Heftdal | 2023 | 1 | 1 | 1 | 0 | 2 | 1 | 1 | 1 | 8 | low |
| Jongkees | 2022 | 0 | 0 | 1 | 0 | 1 | 1 | 0 | 1 | 4 | high |
| Kling | 2023 | 1 | 1 | 1 | 0 | 2 | 1 | 1 | 1 | 8 | low |
| Lamacchia | 2022 | 0 | 0 | 1 | 0 | 1 | 1 | 0 | 1 | 3 | high |
| Lapointe | 2022 | 1 | 1 | 1 | 0 | 1 | 1 | 1 | 1 | 7 | low |
| Lo´ pez-Corte´ s | 2023 | 0 | 0 | 1 | 0 | 1 | 1 | 1 | 0 | 4 | high |
| Lu | 2023 | 0 | 0 | 1 | 0 | 1 | 1 | 1 | 1 | 5 | moderate |
| Malin | 2023 | 0 | 0 | 1 | 0 | 1 | 1 | 1 | 1 | 5 | moderate |
| Moussaoui | 2022 | 1 | 1 | 1 | 0 | 1 | 1 | 1 | 0 | 6 | moderate |
| Park | 2022 | 0 | 1 | 1 | 0 | 1 | 1 | 1 | 1 | 6 | moderate |
| Tan | 2022 | 1 | 1 | 1 | 0 | 0 | 1 | 0 | 1 | 5 | moderate |
| Touizer | 2023 | 1 | 1 | 1 | 0 | 1 | 1 | 1 | 0 | 6 | moderate |
| Vergori(1) | 2022 | 1 | 1 | 1 | 0 | 1 | 1 | 0 | 1 | 6 | moderate |
| Vergori(2) | 2023 | 0 | 0 | 1 | 0 | 0 | 1 | 0 | 1 | 3 | high |
| Vergori(3) | 2022 | 1 | 1 | 1 | 0 | 1 | 1 | 0 | 1 | 6 | moderate |
| wang | 2023 | 1 | 0 | 1 | 0 | 0 | 1 | 1 | 0 | 4 | high |
| Yi | 2023 | 1 | 0 | 1 | 0 | 1 | 1 | 0 | 1 | 5 | moderate |
| Zeng | 2023 | 1 | 0 | 1 | 0 | 1 | 1 | 1 | 1 | 6 | moderate |
| zhan | 2023 | 1 | 1 | 1 | 0 | 2 | 1 | 1 | 1 | 8 | low |
| Zhang | 2023 | 1 | 0 | 1 | 0 | 0 | 1 | 1 | 1 | 5 | moderate |
| Alexandrova | 2023 | 1 | 0 | 1 | 0 | 2 | 1 | 1 | 1 | 7 | low |
| Basso | 2022 | 1 | 0 | 1 | 0 | 0 | 1 | 1 | 1 | 5 | moderate |
| Bessen | 2022 | 0 | 0 | 1 | 0 | 1 | 1 | 1 | 1 | 5 | moderate |
| Chan | 2022 | 1 | 0 | 1 | 0 | 0 | 1 | 1 | 1 | 5 | moderate |
| Cheung | 2023 | 1 | 0 | 1 | 0 | 0 | 1 | 1 | 0 | 4 | high |
| Costiniuk | 2023 | 1 | 0 | 1 | 0 | 1 | 1 | 1 | 0 | 5 | moderate |
| Fidler | 2022 | 0 | 0 | 1 | 0 | 1 | 1 | 1 | 0 | 4 | high |
| Fusco | 2023 | 1 | 0 | 1 | 0 | 0 | 1 | 1 | 1 | 5 | moderate |
| Gianserra | 2022 | 1 | 0 | 1 | 0 | 0 | 1 | 1 | 1 | 5 | moderate |

^#^Low (total score ≥ 7), moderate (total score 5-6), and high (total score ≤ 4) risk of bias.

**Supplementary Table S8. Risk of bias of all included case-control studies using the Newcastle-Ottawa quality assessment scale**

| Author | Selection | | | | Comparability | Exposure | | | Total score | Risk level^#^ |
| --- | --- | --- | --- | --- | --- | --- | --- | --- | --- | --- |
|  | Is the case definition adequate? | Represent ativeness of the cases | Selection of Controls | Definition of Controls | Study controls for antibody positive rate, antibody (the most important factor) Study controls for any additional factor | Ascertainme nt of exposure | Same method of ascertainme nt for cases and controls | NonRespons e rate |  |  |
| Corma-Gómez (2023) | 1 | 1 | 0 | 1 | 1 | 0 | 1 | 0 | 5 | moderate |

^#^Low (total score ≥ 7), moderate (total score 5-6), and high (total score ≤ 4) risk of bias.

**Supplementary Figure S1. Risk of bias of all included studies.**

A B C

Note: The publication bias of studies on immunogenicity among people living with HIV compared with healthy controls after booster dose of COVID-19 vaccine.

Egger’s test. A). immune response rates of SARS-CoV-2 antibodies Egger’s test: Test result: t = -0.96, observed = 29, imputed = 7, p-value = 0.3459; B).Risk ratio of immune response rates of SARS-CoV-2 original strain: Test result: t = -2.66, df = 12, p-value=0.0239, Using the trim-and-fill method to address publication bias, the adjust RR (0.981, 95%CI 0.959-1.002) ; C). immune response rates of SARS-CoV-2 antibodies Omicron variant Egger’s test: Test result: t = -0.82, df = 13, p-value=0.4307.

**Supplementary Figure S2 Sensitivity analysis.**

A B C


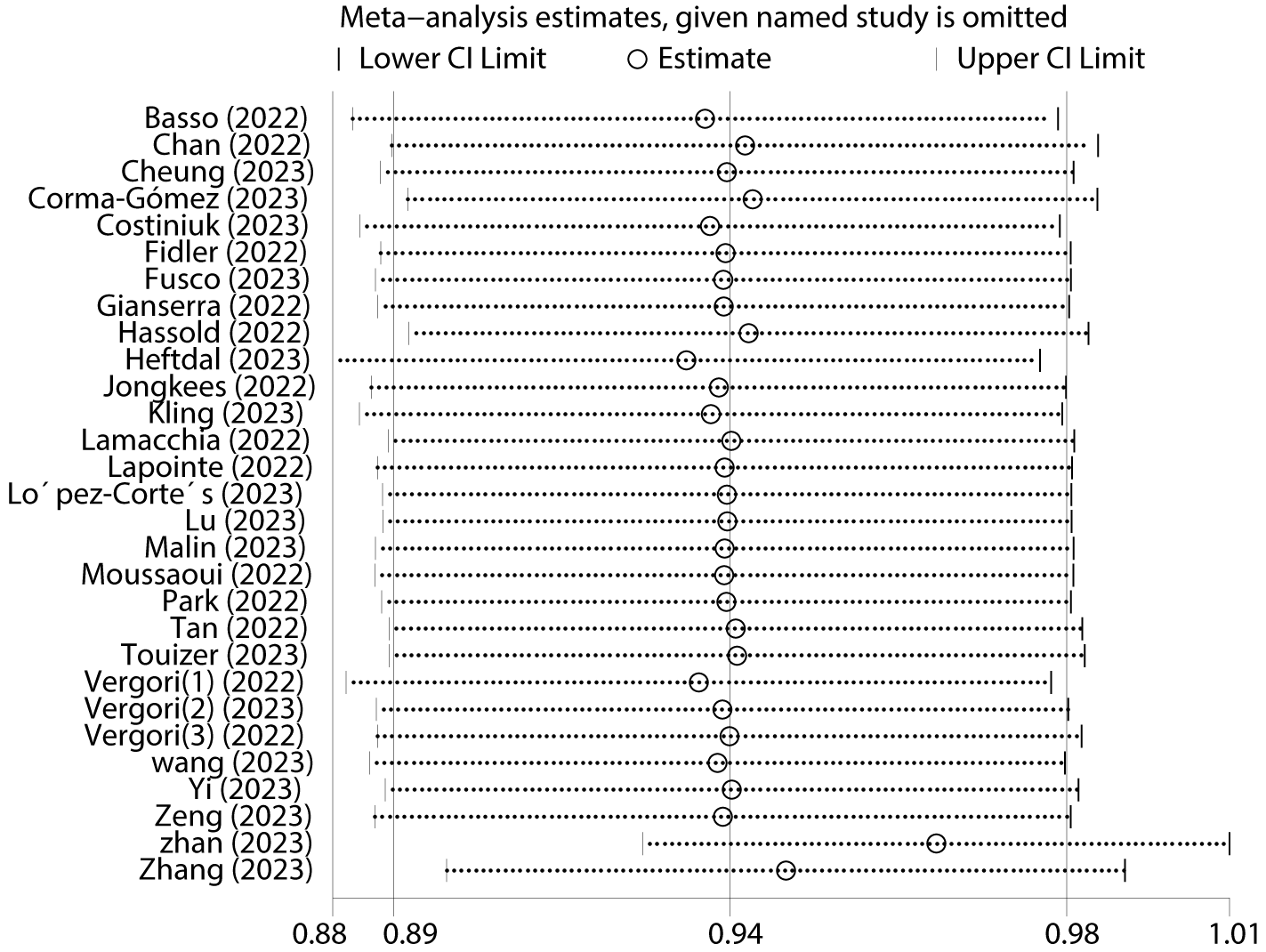

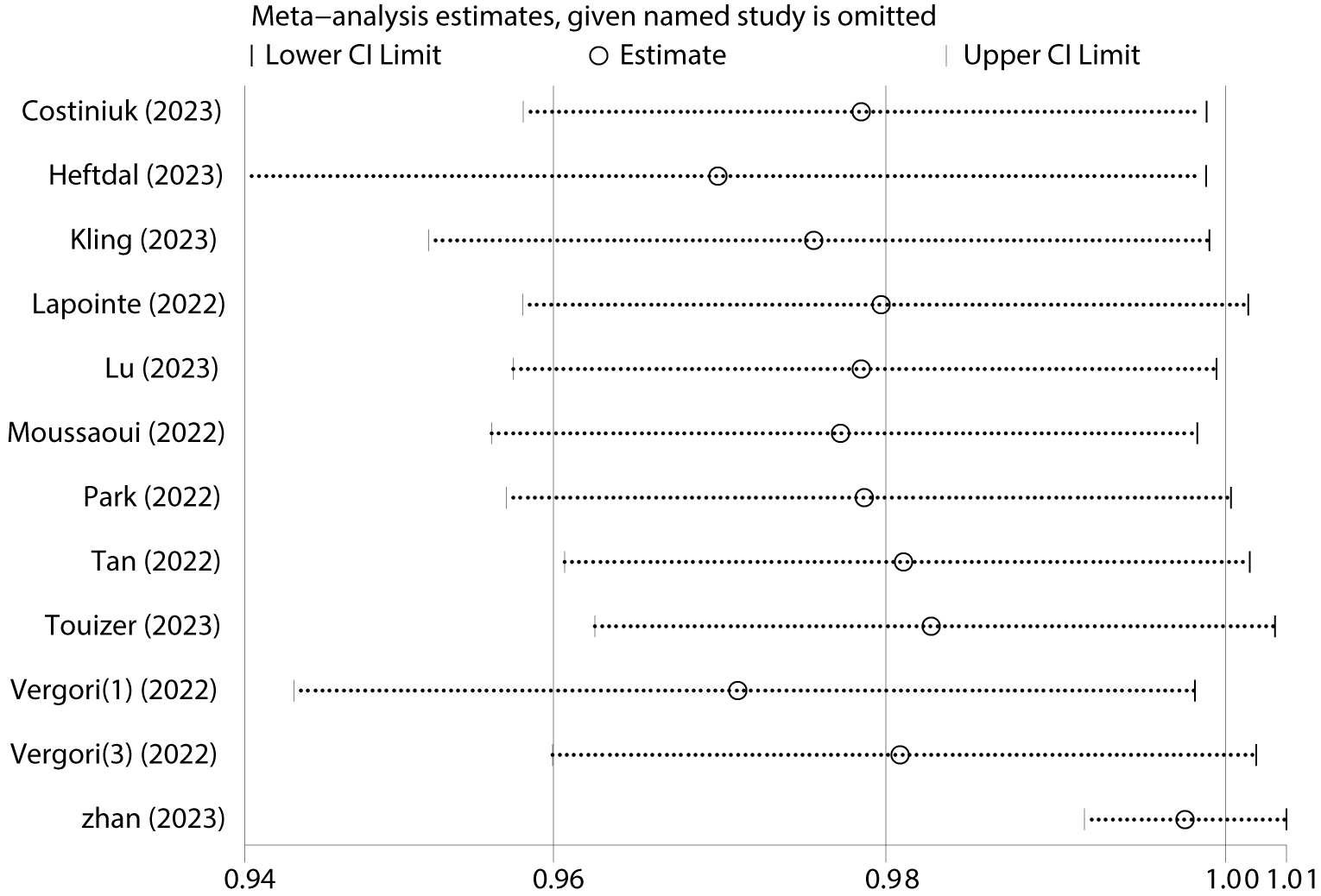

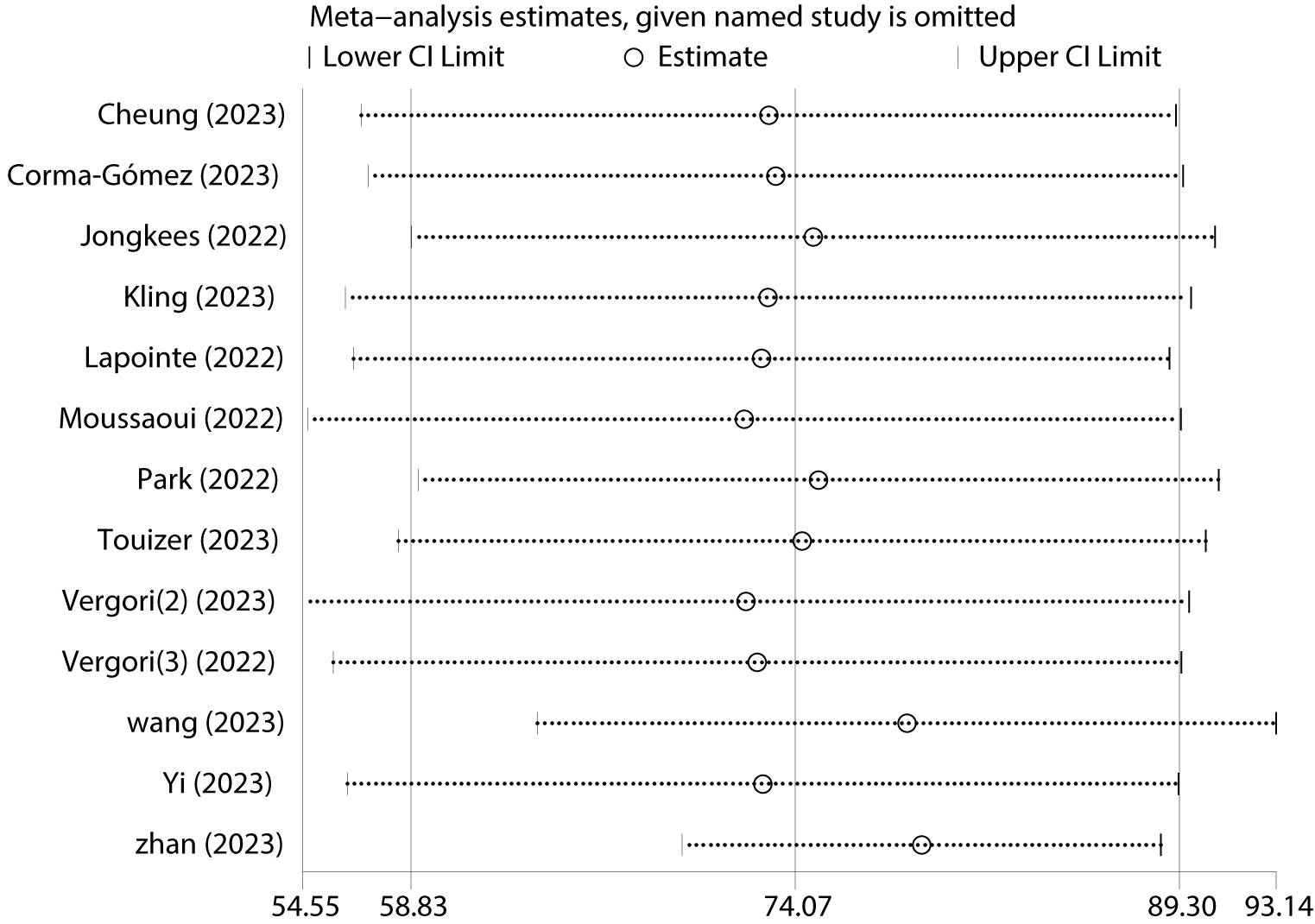


Note. A) Immune response rate of PLWH; B) Immune response rate among PLWH compared to HC; C) SARS-CoV-2 omicron variant antibodies response of PLWH. PLWH, people living with hiv; HC, Healthy Control.
